# Effects of individual differences in text exposure on sentence comprehension

**DOI:** 10.1038/s41598-023-43801-8

**Published:** 2023-10-05

**Authors:** Anastasia Stoops, Jessica L. Montag

**Affiliations:** Psychology Department, University of Illinois Urbana-Champaign, Champaign, 61821 USA

**Keywords:** Human behaviour, Predictive markers

## Abstract

Linguistic experience plays a clear role in accounting for variability in sentence comprehension behavior across individuals and across sentence types. We aimed to understand how individual differences in reading experience predict reading behavior. Corpus analyses revealed the frequencies with which our experimental items appeared in written and spoken language. We hypothesized that reading experience should affect sentence comprehension most substantially for sentence types that individuals primarily encounter through written language. Readers with more text exposure were faster and more accurate readers overall, but they read sentence types biased to written language particularly faster than did readers with less text exposure. We see clear effects of text exposure on sentence comprehension in ways that allow explicit links between written and spoken corpus statistics and behavior. We discuss theoretical implications of effects of text exposure for experience-based approaches to sentence processing.

## Introduction

Linguistic experience—experience with words and sentence structures, has implications for the comprehension of those words and sentence structures. The findings that more frequent structures and structure-word combinations are easier to comprehend are central to many theoretical approaches to psycholinguistics including the classic constraint satisfaction that emerged in the 1990s^1,2^ and continues to be a major component of modern psycholinguistic work^3–7^, including approaches called usage-based or experience-based^8–12^.

The specific aspect of language experience that we investigate here is experience with written language. There are substantial differences between the types of sentences contained in written and spoken language, with written texts containing a greater proportion of rare and complex sentence types, such as passive sentences and sentences containing relative clauses^13,14^. The effect of written language exposure on sentence comprehension is thus both a critical piece of data in support of experience-based accounts of sentence processing, as well as an important source of individual differences in sentence processing.

Accumulating evidence suggests that reading experience may be an important source of individual differences in various aspects of language processing. Reading experience predicts individual differences in vocabulary size^15,16^, lexical decision times^17^, verbal fluency^15^, sentence production^18,19^ and various aspects of sentence comprehension^20–31^. One proposed hypothesis for the observed effects of reading experience on sentence comprehension is greater experience with a subset of sentence types that are more frequent in written language, such as passives^24,31^, relative clauses^20,23,30^, or constructions containing connectives such as *however* or *since*^29,32^. Our approach is to link the statistical properties of speech and text to observed patterns of sentence processing. Individuals with more text exposure should have greater exposure to the types of sentences biased to appear in written language and should show facilitation for those sentences.

We contrast our experience-based approach with syntactic complexity approaches, that suggest that the memory demands on comprehension posed by complex syntactic structures underlie differences in across items and individuals. Under perhaps a straw man version of this approach, reading experience should not affect sentence comprehension because difficulty arises from needing to maintain words or phrases in working memory as the sentence unfolds. Under this account, individual differences in sentence processing are driven by individual differences in memory capacity, which are experience-independent (e.g., Refs.^33,34^). A more nuanced version of this approach (e.g., Refs.^4,35,36^) suggests that both individual differences in experience and memory may uniquely contribute to sentence comprehension. In fact, individual differences approaches to sentence processing often put executive function measures, including memory capacity or verbal IQ in regression models alongside more experience-based measures like vocabulary size or reading experience as independent predictors of behavior^26,27,37–39^.

Our experience-based approach differs from syntactic complexity approaches in two important ways. First, we predict clear frequency by regularity by experience interaction effects such that the effect of experience will be different across sentence types^30,40,41^. For sentence types that are more frequent and more regular (e.g., more similar word order or morphology to meaning mapping to the broader language, such as agents occurring before verbs) written language exposure should have minimal effects on sentence processing. This prediction derives from both non-linear frequency effects in classic learning theory, where learning earlier in training leads to greater changes in behavior than learning later in training^42,43^ and frequency by regularity interactions, where irregular forms benefit more from increased experience than do regular form items^44^. Second, implicit in our approach is that variables such as memory capacity or executive function measures are themselves experience-dependent, as articulated in MacDonald and Christiansen^40^ and Schwering and MacDonald^45^. For example, in line with this idea, some work suggests that the reading span task, used as a measure of working memory capacity, may in fact be an index of language experience-dependent language skill^1,46–48^. We are skeptical of the notion that measures of memory or executive function exist that can be dissociated from experience in the domain in which they are used. This study was not designed to adjudicate between different approaches to sentence processing, because implicit in these debates are deep questions about the nature of human cognition, beyond the scope of any single study. We argue that we can gain significant insight into sentence processing and the cognitive processes that underlie sentence processing by considering an experience-based approach, and considering the different profiles of experience that individuals might gain from written versus spoken language.

We developed a stimulus set consisting of four types of sentences that varied in comprehension difficulty and in their frequencies in written and spoken language: simple active sentences, passive sentences, and sentences containing subject and object relative clauses. To hone our predictions for the effects of reading experience on sentence comprehension, we performed a corpus analysis to discover the frequencies of each sentence type in written and spoken language. To assess sentence comprehension, we recorded participant full-sentence reading times and comprehension question accuracy in a web-based sentence reading task. We then related both reading times and comprehension question accuracy to measures of text exposure.

## Corpus analysis

We predict that text exposure should not predict language comprehension globally, but rather reading experience should lead to better comprehension on sentence types that are more frequent in written language. Individuals with more text exposure should show faster reading times and more accurate sentence comprehension for sentence types that more frequently appear in written language. The goal of this corpus analysis is to determine which sentence types disproportionately appear in written language to understand the aspects of the language environment we expect might change—or not change—with more reading experience.

## Method

Our sentence frequency counts come from a reanalysis of Roland, Dick & Elman^13^, a corpus analysis of the frequencies of a wide range of sentence types in written and spoken corpora. We used the Roland et al. data to calculate frequencies with which our four sentence types, simple transitive sentences, passive sentences, and sentences that contain subject relative clauses (SRCs), and object relative clauses (ORCs), appear in written or spoken language. Our set of simple active sentences do contain some sentence types beyond simple transitive sentences, such as transitive sentences with additional prepositional phrases or conjunctions. Given the available corpus data, and that our sentences indeed all contained transitives, we report the data for simple transitive sentences.

We first calculated frequencies of simple transitive and passive sentences. Roland et al. report passive and simple transitive counts per 100 verb phrases but only overall passive counts. We used the overall corpus size to compute passive counts per million words and use the total passive counts as a reference to convert the simple transitive per 100 verb phrases count to a count of simple transitives per million words.

Computing frequencies for sentences containing relative clauses was slightly more complicated. Roland et al. report separate counts for reduced and full ORCs, so we combine these counts to be more consistent with the counts reported for SRCs, and because we have no reason to believe that the frequencies of both types should not be relevant. Then, due to well-established findings that relative clauses with full embedded noun phrases tend to be harder to comprehend than those with pronominal embedded noun phrases^49–51^, we refined our counts to only include SRCs and ORCs with full embedded noun phrases, not pronouns (e.g., ORC: *the teacher that the student met*; SRC: *the teacher that saw the student).* Roland et al. report numbers of full versus embedded phrase type in SRCs and ORCs in the Brown (written) and Switchboard (Spoken) corpora, but only those with *that* as a relative pronoun, but note that other relative clauses follow a similar pattern. We use these counts of full and embedded NPs to extrapolate counts in the entire corpus.

## Results

**Figure 1:**
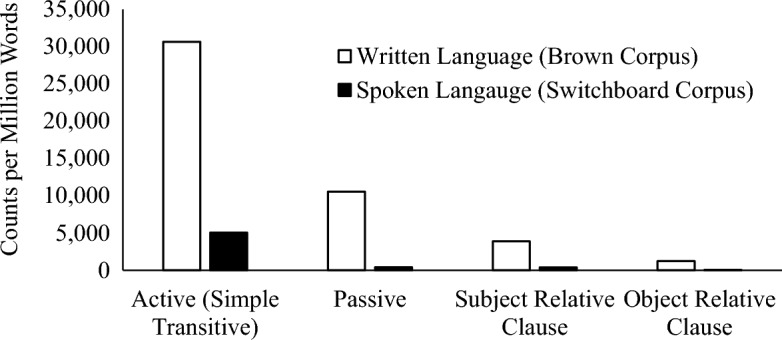
Counts per million words of the four experimental sentence types.

We observe differences in both the absolute frequencies of different sentence types as well as the ratios of frequencies in written and spoken language. Raw counts, counts per million words, and counts per million words including only SRCs and ORCs with full noun phrase embedded noun phrases are shown in Table 1. Table 1 also shows the ratio of the full noun phrase SRC and ORC and passives in written (Brown corpus) and spoken (Switchboard corpus) language. The counts per million words are also plotted in Fig. 1.

**Table 1:** Raw counts, counts per million words, and counts of only subject and object relative clauses with full noun phrase embedded nouns per million words, and ratios of counts in Brown (written) and Switchboard (spoken) corpora.

	Brown			Switchboard			Ratio
	Raw	Per million words	Full NP only	Raw	Per million words	Full NP only	
Active (transitive)	30,641	30,641	NA	7075	5054	NA	6.1
Passive	10,533	10,533	NA	566	404	NA	26.1
SRC	4622	6433	3897	760	543	394	9.9
ORC	2068	2068	1225	870	621	60	20.5

These ratios are imperfect, and the frequency counts may not perfectly reflect the stimuli in our study. For example, if we had been able to limit our corpus counts to only animate-headed SRC and ORCs (as we use in our experimental items) it is possible that the written to spoken language ratios for the SRCs and ORCs would increase. *Animate* headed relative clauses, especially ORCs, with embedded *full noun-phrases* are especially biased to written language^52,53^ so any error associated with ignoring head noun animacy should make our ratios more conservative. That said, these frequencies help us generate broad predictions for behavior based on the experience an individual encounters from spoken and written language.

As is evident from the ratios, all four constructions appear more frequently in written than spoken language. This effect likely reflects that spoken language consists of large proportion of intransitive utterances, as well as many short utterance and sentence fragments^14^. Despite all utterance types appearing more frequently in written texts, the written to spoken ratios vary: The active transitive sentences have the lowest ratio, appearing only about six times more often in text than speech, while SRC appear 10 times more often, ORCs nearly 21 times and passives over 26 times more frequently in speech than text. Notably, passives are the most text-biased construction, despite not containing any embedded clauses, consistent with many previous investigations of passive use^53–57^.

In addition to ratios, the sentence types also vary in overall frequency. The simple transitive sentences are more frequent than the other sentence types, so despite appearing six time more often in written than spoken language, an individual should accumulate considerable experience with these sentences through speech alone. An important question for linking corpus frequencies with predictions for behavior is the role of both the overall and relative frequencies in written and spoken language. We may observe effects on comprehension based on ratios alone, so for all sentences individuals with more text exposure should show facilitation. However, we also expect that raw frequencies will matter as well. The undergraduate participants in our study may have accumulated sufficient experience, particularly with the very regular simple transitive sentences that the extra experience from written language that an avid reader encounters should have little effect on behavior. This prediction stems from the non-linear relationship between experience and behavior^42,43^, and that frequency effects are smaller for more regular items ^(44)^. We expect smaller effects of reading experience for the more globally frequent and regular simple active sentences than for other sentence types which are and overall rare except in written language.

## Main Study: web-based sentence comprehension

The study was pre-registered prior to data collection (https://osf.io/nwk7x).

## Methods

### Participants

All participants were recruited through the Department of Psychology participant pool at the University of Illinois, Urbana-Champaign. All participants gave their informed consent prior to the inclusion in the study. The work was approved by and carried out in accordance with the University of Illinois IRB. 221 native English speakers (mean age: 19; 144 female, 77 male) completed all tasks online.

### Materials

#### Experimental sentences

120 sentences all 12 words each were split in 2 lists in a Latin-square design and presented in a whole sentence self-paced reading fashion. Sentences included 20 simple active sentences, 20 passive main clauses, and 80 sentences containing relative clauses taken from Traxler et al. (Ref.^58^; 40 subject relative clauses (SRC) and 40 object relative clauses (ORC)). Sentences were followed by comprehension questions (See Appendix for a complete list of sentences and questions). Items were pseudorandomized such that no two items of the same kind followed each other. The number of SRC and ORC was doubled relative to simple and passive sentences because SRC and ORC sentences were constructed in pairs (e.g., *The lawyer that the banker…* and *The banker that the lawyer…*) so participants saw only half of the experimental items. The order of the lexical items for the sentential arguments was counterbalanced. For example, if a participant viewed the SRC with the head noun *lawyer* then they would have viewed the ORC with the head noun *banker*. The question phrasing and the order of answer options was counterbalanced as well such that there was an equal number of “yes no” and “no yes” displays and an equal number of “yes” and “no” responses. To avoid participants reading strategically, only half of the comprehension questions for passives probed the understanding of the passive structure proper (e.g., who did what to whom relations) with questions like “Did the cowboy help the nurse?” for a passive sentence like “Yesterday morning, the nurse was helped by the cowboy in ripped jeans.” The other half probed temporal reference “Did the cowboy help the nurse last week?” or the prepositional modifier reference “Did the cowboy wear ripped jeans?”.

1. *Simple sentence*: I went to the store and bought milk, eggs, and green beans.
  Did I go to the library?
  Yes No
2. *Passive main clause*: Yesterday morning, the nurse was helped by the cowboy in ripped jeans.
  Did the cowboy help the nurse last week?
  No Yes
3. *Subject relative clause*: The lawyer that irritated the banker retrieved the paperwork from the office.
  Did the lawyer irritate the banker?
  Yes No
4. *Object relative clause*: The lawyer that the banker irritated retrieved the paperwork form the office.
  Did the banker irritate the lawyer?
  No Yes

### Text exposure surveys

Assessing reading experience is not straightforward. Adults tend to exaggerate reading habits so indirect measures such as Author Recognition Tasks (ART) circumvent social desirability and yield better estimates^59^. The ART is by design an indirect measure of text exposure, and measures logical consequences of text exposure, rather than text exposure itself. However, across multiple languages, the task is predictive of print-related skills, including vocabulary size, reading speed, and word recognition speed^20,60–66^; though perhaps not for L2 speakers: Ref.^67^.

#### Author recognition test

We used an updated version of the Acheson et al.^20^ by Moore and Gordon^66^. See Supplemental Materials for the full survey. This task asks participants to choose real authors from a list of names (60 real, 60 foil authors). Participants received 1 point for a real author and 1 point was subtracted if participants chose a foil name.

#### Reading enjoyment survey

To build converging measures of reading experience, we adapted a survey that measured reading enjoyment in children^68^ to survey reading enjoyment in adults. This survey consists of 10 statements that asked participants to either agree or disagree on a 1 through 7 Likert scale about various reading attitudes to assess participants attitudes and intrinsic motivation associated with reading (e.g., *I enjoy reading; I enjoy receiving books as gifts*). See Supplemental Materials for the full survey. A composite score was computed as the average of all 10 responses. For the questions that probed negative attitudes the scores were flipped to keep positive values at the higher end of the scale.

One motivation for this survey was to obtain a convergent measure of text exposure to complement the ART. Positive attitudes and intrinsic motivation are associated with reading frequency^69^ so we hypothesized assessing attitudes towards reading may allow us to indirectly assess reading experience. A second motivation arises from challenges associated with collecting data online. Even software that locks participants’ screens and prevents them from surfing the internet while performing a task cannot prevent participants from using their phones to look up whether the author is real or not. Assessing reading attitudes may minimize opportunities for participants to “cheat” even if social desirability may become a greater concern.

#### Vocabulary test^70^

Participants were asked to choose a synonym for 40 real English words out of 4 possible variants for each word. Given a suspicious number of perfect or very high scores, it was evident that participants used their phones or other devices to look up correct synonyms for this test. We do not discuss the results further because we believe the results are not reliable.

#### Demographics survey

In-house developed survey that collected basic information pertaining to participants age, gender, SES, and any reading difficulty or dyslexia diagnoses. See Supplemental Materials for full version of the survey.

### Procedure

Participants were given a link after they chose to participate in our experiment through the SONA participant pool administration software. First, they gave consent to participate. Then they were directed to the website that displayed the sentence reading portion of the experiment followed by ART, Vocabulary, Reading Experience survey and basic demographics questionnaire. The experiment was implemented in Ibex farm online software^71^. Ibex farm uses JavaScript and html forms to collect participant responses and response times on the participant’s own computer and uploads participants responses to the server only after participants hit “Finish” button on last page of the experiment. Such approach minimized the response time delays for the reading time measures.

### Data exclusion criteria

Participants who learned English after 5 years of age (N = 40) or reported a history of reading difficulties (N = 23) were excluded from the analyses.

A substantial challenge with online data collection is that it tends to be noisier than data collected in the lab. We developed a pipeline to remove trials and participants that did not likely reflect true reading processes (e.g., “button mashing,” careless clicking, or multitasking during study participation). For the response times, a two-step process was used: first, whole-sentence reading times faster than 1500 ms and slower then 138,000 ms (2.3 min, computed by multiplying 11,500 ms, the highest cut off time used traditionally for one-word-at-a-time self-paced reading studies, by 12 corresponding to 12 words in our sentences) were excluded (3513 data points removed out of the total 21,826 points). Second, the individual whole-sentence reading times were trimmed to cut off 2.5 standard deviations above and below the individual conditional mean (additional 853 data points reducing the dataset to 17,460 total data points). Based on these exclusions the total number of participants was reduced from 280 to 241. Additionally, participants were excluded if previous trimming left less than 50% of items for each of the 4 sentence types (additional 20 participants). Finally, based on our prior observations of individuals completing these tests in person in the lab setting it takes about 5 min maximum to complete the ART test. As a result, we excluded participants who took longer than 300,000 ms (5 min) to complete the test (N = 10 participants). As a result of all the exclusion criteria the final dataset contained 211 participants—64% of the participants who took part in our experiment (343 total participants). These exclusion rates are consistent with other online studies that find 45% to 53% of participants/trials are removed^72^.

### Statistical variables, contrasts, and model fitting considerations

Text exposure surveys (ART, and RE) and sentence type (active, passive, SRC, ORC) were used to predict sentence reading times and comprehension question accuracy. Reading times were analyzed using linear mixed-effects (LME) models, and accuracy results were analyzed with generalized LMM (GLMM) models using the lme4(Version1.1–13; Ref.^73^) and coda^74^ packages in R (Version 3.2.0; Ref.^75^). Three orthogonal contrasts were specified through dummy coding to compare relative clause versus main clause sentences, followed by active versus passive sentences and SRC versus ORC sentences. This coding scheme was preregistered. Three contrasts were defined:

1. Relative Clauses versus Main Clauses (MC vs RC): Active “-1,” Passive “-1,” SRC “1,” and ORC “1”—compares the means of behavioral measures from relative clause sentences (subject and object relative clauses) to the means of the main clause sentences (active and passive sentences).
2. ORC versus SRC (SRC vs ORC): SRC “-1,” ORC “1”, Active “0” and Passive “0” –compares behavioral measures for the object relative clauses to the behavioral measures for the subject relative clauses.
3. Active versus Passive sentences: SRC “0,” ORC “0”, Active “-1” and Passive “1” –compares behavioral measures for the active sentences to the behavioral measures for the passive sentences.

Additionally, as an exploratory analyses after viewing the results, we used treatment contrast where each of the three complex structures were compared to active sentences that served as a baseline. Measure of text exposure (ART and RE scores) were centered and scaled.

**Figure 2:**
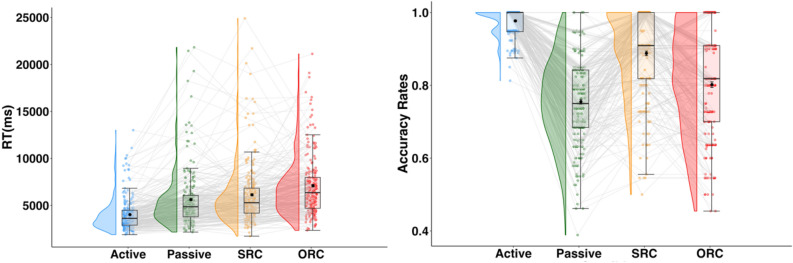
Sentence reading times (left) and comprehension question accuracies (right). Colored dots with grey lines = individual means; black dots with point ranges = conditional means with standard errors.

LME models were fitted to untransformed and log-transformed reading times (See Supplemental materials, Tables 1 and 2 for model results). The results of the transformed and untransformed times were remarkably similar, so we report the untransformed models to facilitate interpretation. We note any significant differences in the pattern of results. The random structure was determined following Barr et al.^76^ maximal fit approach. LME models were fit by restricted maximal likelihood with the Satterthwaite’s method; generalized LME models were fit by maximum likelihood with Laplace approximation. *P*-values were obtained through *summary* function of the *lmerTest* package^77^. The final models for reading times have random slopes for items and participants. The final accuracy models have random slopes for items only due to convergence failure. The exploratory model for accuracy with both ART and RE did not converge with random structure, as a result we fitted this model with regular regression (lm instead of glmer). Response time and accuracy plots in Fig. 2 were inspired by van Langen’s open-source visualizations^78^.

## Results

### Assessments of text exposure

The two assessments of text exposure, the Author Recognition Task (Mean: 13.13, SD = 6.21, Range = − 1–30) and Reading Enjoyment Survey (Mean: 4.27, SD = 1.55, Range = 1.2–7) were only moderately correlated given that they aim to measure the same underlying construct (r = 0.33, p < 0.001); readers with more positive attitudes recognized more real authors. In subsequent analyses, we probe whether the measures each capture variance in our sentence processing measures.

### Reading analyses

Whole-sentence reading time analyses were limited to items on which the participant correctly answered the comprehension question. As expected, participants read the simple sentences faster and more accurately (Fig. 2 and Table 2) than rarer or more syntactically complex sentences. However, relative rankings across the four sentence types for speed and accuracy were not the same. ORC sentences took the longest time to read, followed by SRC, passive, and simple sentences. However, accuracy was the lowest for passive sentences, followed by ORC, then SRC and simple sentences.

**Table 2:** Participants means, sentence counts and standard errors (SE) for reading times and accuracy rates by sentence type.

Sentence type	N correct	Reading times mean (milliseconds)	SE	N overall	Accuracy	SE
Active	3981	4009	59.71	4074	0.98	0.00
Passive	3153	5592	98.59	4176	0.76	0.01
SRC	2233	6121	117.99	2520	0.89	0.01
ORC	2043	7099	126.89	2549	0.80	0.01

### Effects of text exposure on reading time and accuracy

#### Effects of author recognition test

**Figure 3:**
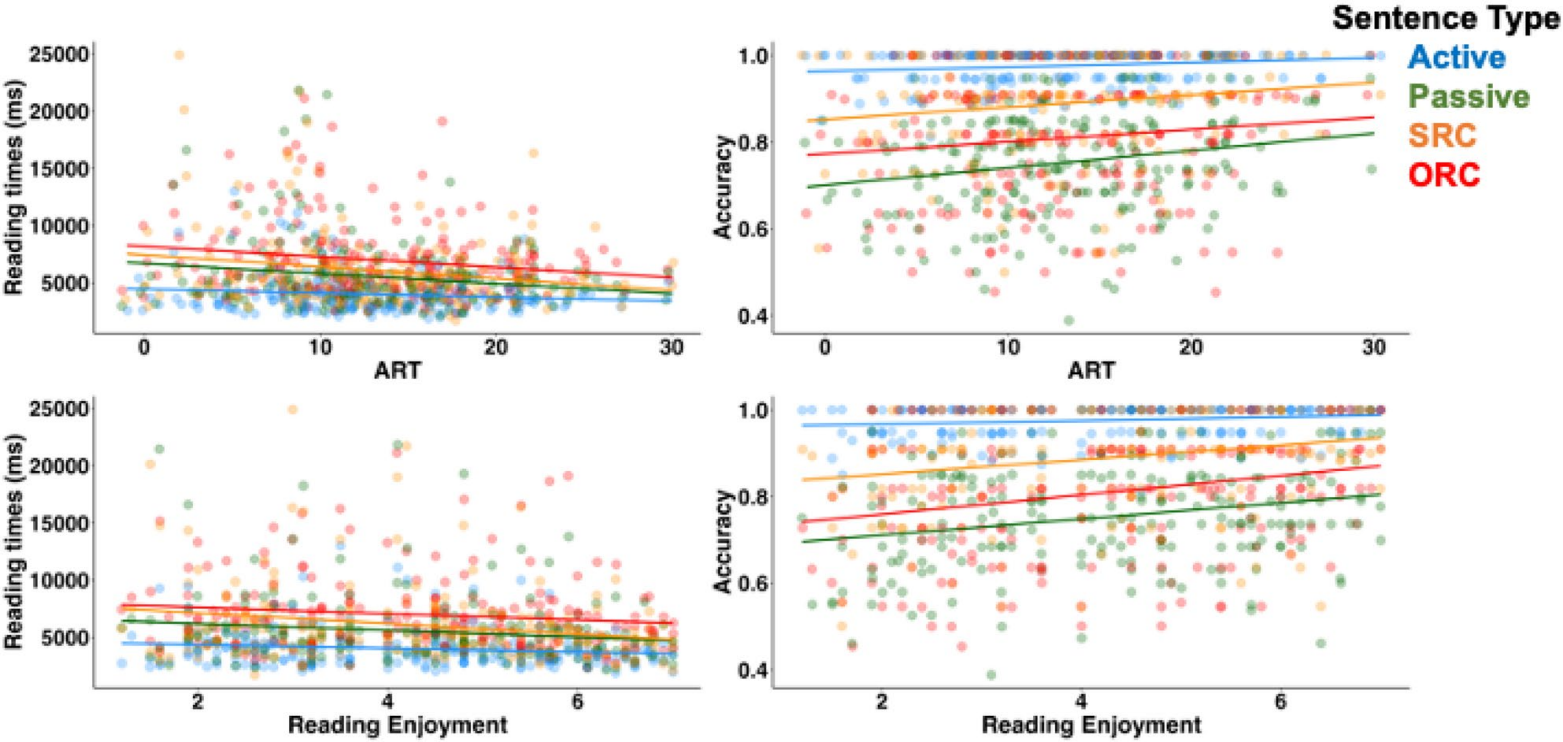
Reading times in milliseconds and comprehension question accuracy rates by ART scores (top row) and RE scores (bottom row) by sentence types. Colored dots = individual means.

To test our key hypothesis, we investigated the how reading experience affected both overall reading times and question accuracy, and how reading times interacted with sentence type. For visualization purposes, Fig. 3 shows the relationship between ART (top row) and RE survey (bottom row) and reading times (first column) and comprehension question accuracy (second column).

Models predicting reading times revealed main effects and an interaction between sentence types and ART score (Table 3, Model 1). All participants read relative clause sentences slower than main clause sentences (main effect of *MC vs RC*), passives slower than active sentences (main effect of *Active vs Passive*), and ORCs slower than SRCs (*SRC vs ORC*). However, the interactions show that participants with higher ART scores read relative clauses (versus main clauses) and passive sentences (versus the active sentences) faster than participants with lower ART scores (ART interaction with *MC vs RC* and *Active vs Passive*). Participants with more text exposure showed smaller differences in reading times for the easier and harder sentences. Log-transformed data revealed a similar pattern of results, except that the main effect for the ART was not reliable (see Supplemental materials, Exhibit A, Table 1, Model 1).

**Table 3:** LME Models predicting the untransformed reading times with condition and ART for pre-registered and exploratory (treatment) contrasts.

Measure	Contrast	b	SE	*t/z*
Model 1: Preregistered dummy coding scheme				
RT ~ MC_RC*ART + A_P*ART + SRC_ORC*ART + (1|Item) + (1|Participant)				
RT in ms	Intercept	**5765.46**	**187.87**	30.688***
	MC vs. RC	**969.14**	**101.29**	9.57***
	Active vs. Passive	**821.65**	**149.54**	5.49***
	SRC vs. ORC	**587.01**	**134.84**	4.35***
	ART	** − 502.95**	**163.77**	− 3.07**
	MC vs. RC: ART	** − 136.07**	**43.52**	− 3.13**
	Active vs. Passive: ART	** − 164.09**	**52.94**	− 3.10**
	SRC vs. ORC: ART	− 36.79	68.74	− 0.54
Model 2: Condition treatment contrasts				
RT ~ A_P*ART + A_SRC*ART + A_ORC*ART + (1|Item) + (1|Participant)				
RT in ms	Intercept	**3974.67**	**261.88**	15.18***
	Active vs. Passive	**1643.29**	**299.09**	5.49***
	Active vs. SRC	**2172.91**	**282.69**	7.69***
	Active vs. ORC	**3346.93**	**285.07**	11.74***
	ART	− 202.78	172.75	− 1.17
	Active vs. Passive: ART	** − 328.18**	**105.88**	− 3.10**
	Active vs. SRC: ART	** − 473.02**	**118.21**	− 4.00***
	Active vs. ORC: ART	** − 399.44**	**122.24**	− 3.27**
Model 3: Preregistered dummy coding scheme				
RT ~ MC_RC*ART + A_P*ART + SRC_ORC*ART + (1|Item) + (1|Participant)				
Accuracy	Intercept	**3.20**	**0.17**	18.85***
	MC vs. RC	** − 1.17**	**0.16**	− 7.24***
	Active vs. Passive	** − 2.85**	**0.39**	− 7.36***
	SRC vs. ORC	** − 0.44**	**0.15**	− 3.01**
	ART	**0.23**	**0.07**	3.11**
	MC vs. RC: ART	0.06	0.06	1.12
	Active vs. Passive: ART	0.11	0.11	0.95
	SRC vs. ORC: ART	0.02	0.04	0.44
Model 4: Condition treatment contrasts				
RT ~ A_P*ART + A_SRC*ART + A_ORC*ART + (1|Item) + (1|Participant)				
Accuracy	Intercept	**4.36**	**0.29**	14.97***
	Active vs. Passive	** − 2.85**	**0.39**	− 7.36***
	Active vs. SRC	** − 1.89**	**0.36**	− 5.32***
	Active vs. ORC	** − 2.77**	**0.35**	− 7.88***
	ART	**0.30**	**0.12**	2.55*
	Active vs. Passive: ART	0.11	0.11	0.95
	Active vs. SRC: ART	0.11	0.13	0.87
	Active vs. ORC: ART	0.15	0.12	1.24

Significant values are in bold.

^p < 0.1; *p < 0.05; **p < 0.01; ***p < 0.001.

When we used our exploratory treatment contrast in the same model (Table 3, Model 2) we get very similar results. All three sentence types were read slower than the active sentences. There was no main effect of the ART but it interacted with all three comparisons. Log-transformed models revealed identical results (Supplemental Material, Exhibit A, Table 1, Model 2).

Across all models, we see a clear effect of text exposure on reading times. We see some evidence that participants who had higher ART score read faster overall, and converging evidence that participants who had higher ART scores were especially faster to read passive, SRC and ORC sentences, the sentence types more frequent in written language, than participants who had lower ART scores.

Generalized LME predicting comprehension question accuracy with ART scores and sentence type revealed only main effects of sentence type and text exposure for both pre-registered and exploratory contrasts (Table 3 Models 3 and 4). Participants were overall less accurate on relative clauses and passive sentences than active sentences and participants with more text exposure were overall more accurate on all sentence types.

#### Effects of reading enjoyment survey

Reading Enjoyment scores showed an identical pattern of effects on reading times as did ART scores for both our preregistered and exploratory model contrasts (Table 4, Model 1 and 2). Log-transformed data revealed identical pattern of results to the raw data with two exceptions: only passive versus active sentences contrast (not the main versus relative clause) yielded significant interaction with the Reading Enjoyment score and both models with pre-registered and exploratory contrasts revealed the main effect of Reading Enjoyment (Supplemental Materials, Exhibit B, Table 2, Model 1 and 2). Effects of Reading Enjoyment scores on comprehension question accuracy were also nearly identical to those of the ART, when using the preregistered contrasts (Table 4 Model 3). However, the same model with the exploratory treatment contrasts converged only with random slopes for items, not participants. Given the potential problems with model fit, we additionally include results from a linear regression model (Table 4, Model 5). We observe no main effect of Reading Enjoyment but see significant interactions between Reading Enjoyment and sentence types such that participants who reported higher degrees of reading enjoyment tend to be more accurate in comprehending all three types of rare or complex sentences relative to the simple sentences than participants who enjoy reading less.

**Table 4:** LME Models for the untransformed reading times, accuracy rates and RE results for pre-registered and exploratory (treatment) contrasts.

Measure	Contrast	b	SE	*t/z*
Model 1: Preregistered dummy coding scheme				
RT ~ MC_RC*RE + A_P*RE + SRC_ORC*RE + (1|Item) + (1|Participant)				
RT in ms	Intercept	**5759.64**	**188.64**	30.53***
	MC vs. RC	**972.47**	**102.43**	9.49***
	Active vs Passive	**820.13**	**151.40**	5.42***
	SRC vs. ORC	**590.06**	**136.15**	4.33***
	RE	** − 486.69**	**165.03**	− 2.95**
	MC vs. RC: RE	** − 119.61**	**43.27**	− 2.76**
	Active vs. Passive: RE	** − 124.09**	**53.42**	− 2.32*
	SRC vs. ORC: RE	− 57.89	67.54	− 0.86
Model 2: Condition treatment contrasts				
RT ~ A_P*RE + A_SRC*RE + A_ORC*RE + (1|Item) + (1|Participant)				
RT in ms	Intercept	**3967.04**	**264.12**	15.02***
	Active vs. Passive	**1640.26**	**302.81**	5.42***
	Active vs. SRC	**2175.01**	**285.90**	7.61***
	Active vs ORC	**3355.13**	**288.30**	11.64***
	RE	− 242.99	174.09	− 1.40
	Active vs. Passive: RE	** − 248.17**	**106.84**	− 2.32*
	Active vs. SRC: RE	** − 421.19**	**117.74**	− 3.58***
	Active vs. ORC: RE	** − 305.41**	**120.80**	− 2.53*
Model 3: Preregistered dummy coding scheme				
Accuracy ~ MC_RC*RE + A_P*RE + SRC_ORC*RE + (1|Item) + (1|Participant)				
Accuracy	Intercept	**3.19**	**0.17**	18.84***
	MC vs. RC	** − 1.15**	**0.16**	− 7.13***
	Active vs. Passive	** − 2.82**	**0.39**	− 7.29***
	SRC vs. ORC	** − 0.44**	**0.15**	− 3.00**
	RE	**0.24**	**0.07**	3.32***
	MC vs. RC: RE	0.03	0.05	0.56
	Active vs. Passive: RE	0.02	0.11	0.15
	SRC vs. ORC: RE	0.01	0.04	0.03
Model 4: Condition treatment contrasts				
Accuracy ~ A_P*RE + A_SRC*RE + A_ORC*RE + (1|Item)				
Accuracy	Intercept	**4.14**	**0.28**	15.03***
	Active vs. Passive	** − 2.72**	**0.37**	− 7.36***
	Active vs. SRC	** − 1.84**	**0.34**	− 5.39***
	Active vs. ORC	** − 2.68**	**0.34**	− 7.97***
	RE	**0.22**	**0.10**	2.10*
	Active vs. Passive: RE	0.02	0.05	0.86
	Active vs. SRC: RE	0.02	0.05	0.40
	Active vs. ORC: RE	0.01	0.04	0.14
Model 5 (lm)				
Accuracy ~ A_P*RE + A_SRC*RE + A_ORC*RE				
Accuracy	Intercept	**0.98**	**0.01**	184.95***
	Active vs. Passive	** − 0.22**	**0.01**	− 29.91***
	Active vs. SRC	** − 0.09**	**0.01**	− 10.66***
	Active vs. ORC	** − 0.18**	**0.01**	− 20.67***
	RE	0.01	0.01	0.93
	Active vs. Passive: RE	**0.03**	**0.01**	3.50***
	Active vs. SRC: RE	**0.02**	**0.01**	2.29*
	Active vs. ORC: RE	**0.03**	**0.01**	3.79***

Significant values are in bold.

^p < 0.1; *p < 0.05; **p < 0.01; ***p < 0.001.

#### Variance accounted for by ART and reading enjoyment scores

In exploratory follow-up analyses, ART and Reading Enjoyment scores were put in the same model with the pre-registered contrasts and exploratory treatment contrasts to investigate whether the two measures of text exposure accounted for similar or different sources of variance in reading times and comprehension accuracy. Full models are presented in Supplemental Materials (Exhibit C). Despite the relatively low correlation between the two measures of text exposure (r = 0.33) and that each independently predicted reading times, we found no evidence that the inclusion of both ART and Reading Enjoyment in a model predicting reading times improved fit over including only a single predictor. Though we find some evidence that ART and RE may account for non-overlapping variance in comprehension question accuracy, given potential issues with model convergence and data sparsity, we cannot strongly draw this conclusion. We delegate it to future studies to investigate the sources of similarities and differences between ART and Reading Enjoyment further.

## Discussion

In a web-based experiment, we found differences in the speed with which participants read, and accuracy with which participants answered comprehension questions about four sentence types: simple active sentences, passive sentences and sentences containing subject and object relative clauses. Crucially, we found robust individual differences such that individuals with more text exposure read passive sentences and the sentences containing relative clauses more quickly and overall answered comprehension questions more accurately than participants with less text exposure.

Our key hypothesis was that text exposure should interact with sentence type. Text exposure should not uniformly affect sentence comprehension but rather we should see the strongest effects for the sentence types for which reading should most dramatically affect one’s linguistic experience. We do see some evidence of main effects of text exposure on reading speed and clear evidence of main effects of text exposure on comprehension question accuracy. However, we also found sentence type by reading experience interactions. Individuals with more text exposure were faster particularly for the passive sentences and sentences containing SRC and ORC that individuals should encounter relatively more frequently from written language. For participants with more text exposure, reading times for the rarer, written-language biased sentences approached those of the simple active sentences. We found weaker evidence for similar interactions in comprehension question accuracy. It is possible we might have found stronger interactions had there been greater variability in the text exposure of our participants (all were enrolled undergraduate students), or this lack of an interaction might stem from different trials being included in reading time versus accuracy analyses (incorrect responses were excluded from reading time analyses) or some other difference between the processes that underlie reading times versus reading accuracies.

Our results have clear implications for experience-based accounts of sentence processing. Experience interacts with sentence type in predictable ways. We see stronger effects of text exposure on items for which we expect that experience should come predominantly from written language. A potential concern is whether the interaction between sentence type and text exposure reflects a true interaction or is an artifact of a floor effect in the simple active sentence. We argue that this “floor effect” may in fact be evidence of experience-based sentence processing. Our college-aged participants are sufficiently experienced, through both speech and text, with simple active sentences such that additional experience through higher rates of text exposure had little effect on behavior. It then follows that for less experienced readers, like children or adolescents, we would not expect a floor effect, but rather see robust effects of text exposure on even the simple active sentences.

**Figure 4:**
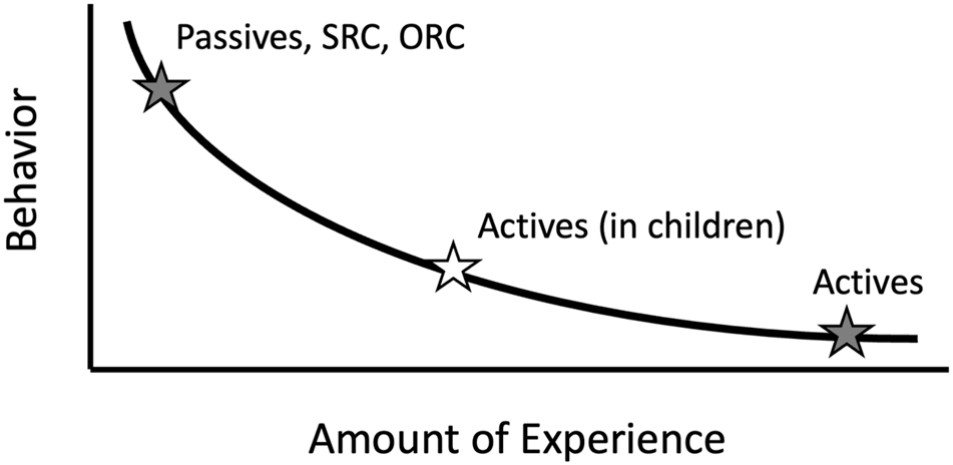
A visualization of our hypothesized relationship between language experience and behavior.

This hypothesis derives from notions in classic learning theory^79–81^ as well as error-driven learning theories^82–84^, that early in learning, learning proceeds more quickly than it does later, with a consequence on observable behavior like that depicted abstractly in Fig. 4 At overall low rates of experience with a sentence structure, such as with text-biased passives indicated by the grey star, reading may be slow or inaccurate. With these low-experience structures, a small amount of extra experience has a large effect on behavior—speeding up reading times or increasing question accuracy. Moving rightward on the curve reflects structures with which participants have more experience, so differences in extra experience (x-axis) lead to lower measurable changes in behavior. Active sentences in adults are very far to the right of the curve, and so additional experience has little effect on behavior. This asymptotic effect of behavior given experience is why we see little effect of text exposure on active sentences. However, in children, who have both less experience with spoken language and substantially less experience with written language, we expect actives to be higher on the curve, such that individual differences in text exposure, should be associated with a measurable effect on behavior. Our approach provides both a coherent account of the observed data and makes important predictions for patterns of behavior in less experienced readers (e.g., children, adolescents) as well expected patterns of behavior in other sentence types that may appear with different frequencies in written and spoken language.

In addition to the non-linear frequency effects described above, reading time differences can also be driven by regularity effects. As learning progresses, differences emerge in the regularity of the mappings between sentence types and other associated patterns, such as the relationship between word order and semantic roles. For example, across many English sentence types, agents typically precede verbs and -ed morphology typically maps to a past tense marker. However, some sentences violate these broad tendencies: In passive sentences and in object relative clauses, agents follow verbs, and in some passives -ed maps to a passive marker (was consumed vs. was eaten). More regular mappings are learned more easily or thoroughly, facilitating comprehension of sentences that are consistent with these patterns. In many domains, it has been shown that these frequency and regularity effects have a further interaction effect^44^. In sentence comprehension, structures that are frequent but not regular, or structures that are regular but not frequent, are both learned well. It is structures that are neither frequent nor regular that the farthest to the left on the curve in Fig. 4, and that benefit most from additional exposure. Neural network models^40,85^, training studies with humans^41^, and other behavioral studies^30^ identify frequency by regularity interactions in sentence comprehension. The greater effects of text exposure for passive sentences and relative clauses likely arise from not only non-linear frequency effects, but subsequent frequency by regularity interactions as well.

The joint contribution of non-linear frequency effects and frequency by regularity interactions speaks to the importance of formalizing the complex association between input and behavior. Both can be formalized in a variety of different models and can arise from different mechanisms. For example, nonlinear frequency effects can arise directly from direct changes in learning rates that are higher early in learning and lower later in learning^86^. A second mechanism arises out of the nonlinear activation function present in most learning models. For example, a model using a sigmoid activation function will see the greatest changes in learning early on, when the middle range of that function, and less change when the model is at one of its asymptotic extremes^87^. A third mechanism by which learning starts fast and then slows down is an emergent property of error-driven learning (like the Delta-rule, backpropagation, or the Rescorla-Wagner model), where weight changes in a model are proportional to the amount of error in the model^88–91^. In these models, nonlinear frequency and regularity effects emerge from the learning itself, as the model forms generalizations over frequent or regular input patterns and applies those patterns to subsequent trials. All three of these approaches make different assumptions about human learning mechanisms, but all highlight the centrality of the input or training set to understanding behavior.

This work adds to a body of work emphasizing the role of experience in sentence processing, consistent with many experience-based accounts^8,9,11,12^. Further, we suggest that for many adults, text exposure, specifically, may capture variability in language experience. This work builds upon existing effects of text exposure on the comprehension of the sentences we test here, including subject and object relative clauses^20,23,26,30^ and passives^31,92^ This work cannot speak to the relative role of language experience versus memory or executive function effects on sentence processing—implicit in these debates is the in-principle plausibility of an experience-independent measure of memory or executive function, and other deep assumptions about cognition. Rather, we see this work as highlighting the potentially large amount of variance, across items and individuals, that can be accounted for by language experience.

In this work, we also attempted to establish the utility of a Reading Enjoyment survey that may corroborate or complement the commonly used Author Recognition Task (ART). We found that the ART and Reading Enjoyment survey generally accounted for overlapping variance despite being only moderately correlated themselves. Putting both measures of text exposure in a single model did not improve model fit. Larger samples may be necessary to more clearly understand the overlapping or non-overlapping aspects of text exposure that ART and Reading Enjoyment may capture. However, we identify a clear disadvantage of the ART in web-based studies: participants seem to use their phones or other devices to look up author names. We saw similar evidence of this device use in our Shipley vocabulary scores, which were unrealistically high and as such, unusable. While Reading Enjoyment surveys may not replace the ART given the ART’s long history of successful use, web-based data collection may want to consider other means, like the Reading Enjoyment survey of assessing text exposure to complement the ART. Alternatively, adding a time limit on the ART display or presenting author names one at a time might discourage participants use of other devices during online study participation.

One question that remains is why we found such different rankings across our four sentence types for reading times and accuracies. Passive sentences were the second-fastest read sentence (after active sentences) but were the least accurately comprehended. There are several potential explanations. First, it is possible that online (reading times) versus offline measures (comprehension question accuracy) assess subtly different aspects of sentence processing or individual differences. For example, James et al. (2018) finds effects of individual differences in only offline, not online, measures. In a related vein, because reading times were computed only for trials on which participants correctly answered the comprehension question, there may be different compositions of and sources of variability in the reading time and question accuracy measures.

Second, rather unintuitively, given that passives do not contain embedded clauses and prescriptive advice to avoid passives in writing, passives are remarkably biased to appear in written language. In our corpus analysis, passives were more text-biased than the SRC and ORC containing sentences. So perhaps the question ought to be not why passives were so poorly comprehended, but why were they read so quickly. Previous work also finds low rates of comprehension accuracy for passive sentences^93–95^ but no difficulty or even facilitation on online processing measures^96–99^. These results could be interpreted as a replication of the “good-enough” processing account^93,100^, that suggests that passive sentences are read quickly perhaps because they are interpreted as actives.

Passive sentences may be particularly prone to misanalysis because of morphological features of the English passive that provides imperfect cues to a passive constriction—they are “irregular.” Relative clauses all contained the complementizer “that” with full noun phrases: both are strong, unambiguous cues for a subordinate clause. In English, passive sentences have much weaker cues to their sentence type, at the verb and participle up to the “by”-phrase. In English morphology “was” and “ed” are not exclusive to passive sentences and passives can be interpreted as other sentence types as the sentence is unfolding, or even as a copula construction and an adjective up until the by-phrase as in the sentence “*The nurse was surprised.*” Passive utterances may be read quickly because they are particularly prone to misanalysis. Evidence for misanalyses in other sentence types is primarily reported in off-line accuracy measures (Ref.^100–102^ but see Ref.^103^ for both online and off-line effects) just as we see with our passive sentences. Future work can also clarify how the ability to use the imperfect morphological cues to the passive may change with experience (in essence, frequency by regularity by experience effects), to allow us to understand more precisely what individuals with more or less text exposure may be doing during online and offline sentence processing.

This work provides evidence of effects of text exposure on sentence processing. Moreover, this work suggests pathways by which corpus statistics of spoken and written language could be used to further explore individual differences in language comprehension. The hypothesized pathways introduce clear experimental hypotheses as well as avenues of formal modeling to better understand the links between input and language behavior. Future work may also benefit from finer-grained measures of sentence processing, including word-by-word reading times which allow experimenters to understand the locus of comprehension difficulty, as well as eye tracking measures that can distinguish between earlier and later measures of processing (e.g., first fixation vs. regressions) that can help us better understand the time course of sentence comprehension processes.

### Supplementary Information

Supplementary Tables.

Supplementary Information.

## Data Availability

Stimuli, results, and analytical scripts are available on OSF repository https://osf.io/vct7s/.
