# Supplementary material for: Effects of individual differences in text exposure on sentence comprehension
Source: Sci Rep. 2023 Oct 5;13:16812. doi: 10.1038/s41598-023-43801-8 (PMC10556088; doi:10.1038/s41598-023-43801-8)
Supplement: Supplementary file 1 — Supplementary Tables. [file 41598_2023_43801_MOESM1_ESM.pdf]

Supplemental Materials

Exhibit A

Measure	Contrast	b	SE	*t/z*
**Model 1: Preregistered dummy coding scheme**				
RT ~ MC_RC*ART + A_P*RT + SRC_ORC*ART +(1|Item) + (1|Participant)				
RT in log	Intercept	**8.44**	**2.53**	**333.86*** **
	MC vs RC	**1.71**	**1.40**	**12.18*** **
	Active vs. Passive	**1.50**	**2.13**	**7.00*** **
	SRC vs ORC	**8.61**	**1.79**	**4.80*** **
	ART	*-4.12*	*2.14*	*-1.92^*
	MC vs RC: ART	**-1.02**	**4.37**	**-2.34***
	Active vs. Passive: ART	**-1.59**	**5.30**	**-2.99*** *
	SRC vs ORC: ART	1.10	6.91	.16
**Model 2: Condition treatment contrasts**				
RT ~ Condition*ART +(1|Item) + (1|Participant)				
RT in log	Intercept	**8.11**	**3.66**	**221.77*** **
	Active vs Passive	**3.00**	**4.27**	**7.00*** **
	Active vs SRC	**4.05**	**3.93**	**10.32*** **
	Active vs ORC	**5.77**	**3.96**	**14.60*** **
	ART	-1.51	2.21	-.68
	Active vs Passive: ART	**-3.17**	**1.06**	**-2.99*** *
	Active vs SRC: ART	**-3.74**	**1.19**	**-3.15*** *
	Active vs ORC: ART	**-3.52**	**1.23**	**-2.87*** *

Table 1. LME Models for the log transformed reading times with ART. Note: ^p<.1;*p<.05; **p<.01; ***p<.001

Exhibit B

Measure	Contrast	b	SE	*t/z*
**Model 1: Preregistered dummy coding scheme**				
RT ~ MC_RC*RE + A_P*RE + SRC_ORC*RE +(1|Item) + (1|Participant)				
RT in log	Intercept	**8.45**	**2.51**	**336.14*** **
	MC vs RC	**1.71**	**1.41**	**12.12*** **
	Active vs. Passive	**1.49**	**2.15**	**6.95*** **
	SRC vs ORC	**8.63**	**1.80**	**4.79*** **
	RE	**-6.43**	**2.13**	**-3.01*** *
	MC vs RC: RE	-6.19	4.33	-1.43
	Active vs. Passive: RE	**-1.41**	**5.35**	**-2.63*** *
	SRC vs ORC: RE	-7.30	6.77	-.11
**Model 2: Condition treatment contrasts**				
RT ~ Condition*RE +(1|Item) + (1|Participant)				
RT in log	Intercept	**8.12**	**3.66**	**221.63*** **
	Active vs Passive	**2.99**	**4.23**	**6.95*** **
	Active vs SRC	**4.05**	**3.95**	**10.25*** **
	Active vs ORC	**5.78**	**3.98**	**14.52*** **
	RE	**-4.40**	**2.20**	**-2.00***
	Active vs Passive: RE	**-2.82**	**1.07**	**-2.63*** *
	Active vs SRC: RE	**-2.57**	**1.18**	**-2.18***
	Active vs ORC: RE	**-2.72**	**1.21**	**-2.25***

Table 2. LME Models for the log transformed reading times with RE. Note: ^p<.1;*p<.05; **p<.01; ***p<.001

Exhibit C

Measure	Contrast	b	SE	*t/z*
**Model 1: Preregistered dummy coding scheme**				
RT ~ MC_RC*ART + A_P* ART + SRC_ORC* ART +MC_RC*RE + A_P*RE + SRC_ORC*RE ++ (1|Item) +(1|Participant)				
RT in ms	Intercept	**5760.38**	**187.29**	**30.76*** **
	MC vs RC	**969.19**	**101.61**	**9.54*** **
	Active vs. Passive	**820.40**	**150.05**	**5.47*** **
	SRC vs ORC	**588.53**	**135.21**	**4.35*** **
	ART	**-373.86**	**175.89**	**-2.13***
	RE	*-345.53*	*177.09*	*-1.95^*
	MC vs RC: ART	**-107.05**	**46.46**	**-2.30***
	Active vs. Passive: ART	**-137.64**	**56.59**	**-2.43***
	SRC vs ORC: ART	-19.69	73.23	-.27
	MC vs RC: RE	*-82.30*	*46.18*	*-1.78^*
	Active vs. Passive: RE	-74.81	57.10	-1.31
	SRC vs ORC: RE	-51.88	71.93	-.72
RT in log	Intercept	**8.44**	**2.51**	**336.32*** **
	MC vs RC	**1.71**	**1.41**	**12.15*** **
	Active vs. Passive	**1.49**	**2.14**	**6.99*** **
	SRC vs ORC	**8.63**	**1.80**	**4.80*** **
	ART	-1.99	2.28	-.87
	RE	**-5.68**	**2.30**	**-2.46***
	MC vs RC: ART	*-9.12*	*4.66*	*-1.96^*
	Active vs. Passive: ART	**-1.25**	**5.67**	**-2.20***
	SRC vs ORC: ART	-1.59	7.37	-.22
	MC vs RC: RE	-3.00	4.63	-.65
	Active vs. Passive: RE	*-9.62*	*5.72*	*-1.68*
	SRC vs ORC: RE	-1.22	7.21	-.17
**Model 2: Condition treatment contrasts**				
RT ~ Condition*ART + Condition*RE + (1|Item) +(1|Participant)				
RT in ms	Intercept	**3970.79**	**261.92**	**15.16*** **
	Active vs. Passive	**1640.81**	**300.10**	**5.47*** **
	Active vs. SRC	**2170.24**	**283.56**	**7.65*** **
	Active vs. ORC	**3347.31**	**285.97**	**11.71*** **
	ART	-129.16	185.44	-.70
	RE	-188.42	186.72	-1.00
	Active vs. Passive: ART	**-275.29**	**113.19**	**-2.43***
	Active vs. SRC: ART	**-371.43**	**126.01**	**-2.95****
	Active vs. ORC: ART	**-332.05**	**13-.63**	**-2.54***
	Active vs. Passive: RE	-149.63	114.19	-1.31
	Active vs. SRC: RE	**-291.29**	**125.48**	**-2.32***
	Active vs. ORC: RE	-187.53	129.07	-1.45
RT in log	Intercept	**8.12**	**3.58**	**227.05*** **
	Active vs. Passive	**3.00**	**4.20**	**7.13*** **
	Active vs. SRC	**4.04**	**3.87**	**10.44*** **
	Active vs. ORC	**5.75**	**3.90**	**14.75*** **
	ART	-1.50	2.29	-.07

	RE	-3.85	2.30	-1.67^
	Active vs. Passive: ART	-2.13	1.10	-1.93^
	Active vs. SRC: ART	**-3.23**	**1.23**	**-2.63****
	Active vs. ORC: ART	**-2.87**	**1.28**	**-2.25***
	Active vs. Passive: RE	-2.09	1.11	-1.89^
	Active vs. SRC: RE	-1.30	1.22	-1.06
	Active vs. ORC: RE	-1.54	1.26	-1.23

Model 3: Preregistered dummy coding scheme
Accuracy ~ MC_RC*ART + A_P* ART + SRC_ORC* ART +MC_RC*RE + A_P*RE + SRC_ORC*RE + (1|Item)

Accuracy	Intercept	**3.03**	**.16**	**19.43*****
	MC vs RC	**-1.14**	**.16**	**-7.36*****
	Active vs. Passive	**-2.74**	**.37**	**-7.40*****
	SRC vs ORC	**-.42**	**.14**	**-3.01****
	ART	**.15**	**.06**	**2.54***
	RE	**.19**	**.06**	**3.22****
	MC vs RC: ART	.08	.06	1.36
	Active vs. Passive: ART	.13	.12	1.06
	SRC vs ORC: ART	.02	.05	.47
	MC vs RC: RE	.06	.06	.91
	Active vs. Passive: RE	.04	.13	.35
	SRC vs ORC: RE	.01	.04	.18

Model 4: Condition treatment contrasts
Accuracy ~ Condition*ART + Condition*RE + (1|Item)

Accuracy	Intercept	**.98**	**.01**	**185.00*****
	Active vs. Passive	**-.22**	**.01**	**-29.94*****
	Active vs. SRC	**-.10**	**.01**	**-10.65*****
	Active vs. ORC	**-.18**	**.01**	**-20.65*****
	ART	.01	.01	.91
	RE	.00	.01	.54
	Active vs. Passive: ART	.01	.01	1.37
	Active vs. SRC: ART	.00	.01	.32
	Active vs. ORC: ART	.00	.01	.50
	Active vs. Passive: RE	**.02**	**.01**	**2.79****
	Active vs. SRC: RE	**.02**	**.01**	**2.10***
	Active vs. ORC: RE	**.03**	**.01**	**3.43*****

Table 3. LME Models for the raw and log transformed reading times and accuracy rates with ART and RE tests included in the same model with the pre-registered and exploratory (treatment) contrasts. Note: ^p<.1; *p<.05; **p<.01; ***p<.001 Since accuracy model with exploratory contrasts did not converge with random structure we present here the results of logistic regression model.
