# Supplementary material for: Effects of individual differences in text exposure on sentence comprehension
Source: Sci Rep. 2023 Oct 5;13:16812. doi: 10.1038/s41598-023-43801-8 (PMC10556088; doi:10.1038/s41598-023-43801-8)
Supplement: Supplementary file 2 — Supplementary Information. [file 41598_2023_43801_MOESM2_ESM.pdf]

Appendix
Stimuli

Note: Correct response is bolded

Item ID	Sentence	Question	Answer1	Answer2	SentenceType	List
1	The lawyer that the banker irritated retrieved the paperwork from the office.	Did the banker irritate the lawyer?	**Yes**	No	Object	1
1	The banker that irritated the lawyer played tennis every Saturday and Sunday.	Did the banker irritate the lawyer?	**Yes**	No	Subject	1
1	The banker that the lawyer irritated played tennis every Saturday and Sunday.	Did the lawyer irritate the banker?	**Yes**	No	Object	2
1	The lawyer that irritated the banker retrieved the paperwork from the office.	Did the lawyer irritate the banker?	**Yes**	No	Subject	2
2	The child that the babysitter chased squealed with delight at the game.	Did the babysitter squeal?	Yes	**No**	Object	1
2	The babysitter that chased the child tripped over the toy dump truck.	Did the child trip?	Yes	**No**	Subject	1
2	The babysitter that the child chased tripped over the toy dump truck.	Did the child trip?	Yes	**No**	Object	2
2	The child that chased the babysitter squealed with delight at the game.	Did the babysitter squeal?	Yes	**No**	Subject	2
3	The secretary that the businessman married enjoyed playing soccer on the weekends.	Did the secretary enjoy playing soccer?	**Yes**	No	Object	1
3	The businessman that married the secretary invited the bookkeeper to the party.	Was the bookkeeper invited to the party?	**Yes**	No	Subject	1
3	The businessman that the secretary married invited the bookkeeper to the party.	Was the bookkeeper invited to the party?	**Yes**	No	Object	2
3	The secretary that married the businessman enjoyed playing soccer on the weekends.	Did the secretary enjoy playing soccer?	**Yes**	No	Subject	2
4	The writer that the photographer complimented worked for a famous national magazine.	Did the writer compliment the photographer?	Yes	**No**	Object	1
4	The photographer that complimented the writer captured the news as it happened.	Did the writer compliment the photographer?	Yes	**No**	Subject	1
4	The photographer that the writer complimented captured the news as it happened.	Did the photographer compliment the writer?	Yes	**No**	Object	2
4	The writer that complimented the photographer worked for a famous national magazine.	Did the photographer compliment the writer?	Yes	**No**	Subject	2
5	The hiker that the fisherman passed got lost and was eventually rescued.	Did the hiker get lost?	**Yes**	No	Object	1

5	The fisherman that passed the hiker carried lots of heavy camping gear.	Did the fisherman carry the gear?	**Yes**	No	Subject	1
5	The fisherman that the hiker passed carried lots of heavy camping gear.	Did the fisherman carry the gear?	**Yes**	No	Object	2
5	The hiker that passed the fisherman got lost and was eventually rescued.	Did the hiker get lost?	**Yes**	No	Subject	2
6	The plumber that the electrician helped retired after twenty years of work.	Did the plumber help the electrician?	Yes	**No**	Object	1
6	The electrician that helped the plumber knew where the spare was kept.	Did the plumber help the electrician?	Yes	**No**	Subject	1
6	The electrician that the plumber helped knew where the spare was kept.	Did the electrician help the plumber?	Yes	**No**	Object	2
6	The plumber that helped the electrician retired after twenty years of work.	Did the electrician help the plumber?	Yes	**No**	Subject	2
7	The golfer that the caddy liked played ice hockey during the winter.	Did the golfer play ice hockey during the winter?	**Yes**	No	Object	1
7	The caddy that liked the golfer suggested a new restaurant for lunch.	Did the caddy suggest a new restaurant?	**Yes**	No	Subject	1
7	The caddy that the golfer liked suggested a new restaurant for lunch.	Did the caddy suggest a new restaurant?	**Yes**	No	Object	2
7	The golfer that liked the caddy played ice hockey during the winter.	Did the golfer play ice hockey during the winter?	**Yes**	No	Subject	2
8	The officer that the hunter saw cautioned against staying in the woods.	Did the hunter caution the officer?	Yes	**No**	Object	1
8	The hunter that saw the officer ran off into the dense forest.	Did the officer run into the forest?	Yes	**No**	Subject	1
8	The hunter that the officer saw ran off into the dense forest.	Did the officer run into the forest?	Yes	**No**	Object	2
8	The officer that saw the hunter cautioned against staying in the woods.	Did the hunter caution the officer?	Yes	**No**	Subject	2
9	The historian that the freshman criticized felt really bad after the incident.	Did the historian criticize the fisherman?	**Yes**	No	Object	1
9	The freshman that criticized the historian preferred reading about the Roman Empire.	Did the fisherman criticize the historian?	**Yes**	No	Subject	1
9	The freshman that the historian criticized preferred reading about the Roman Empire.	Did the fisherman criticize the historian?	**Yes**	No	Object	2
9	The historian that criticized the freshman felt really bad after the incident.	Did the historian criticize the fisherman?	**Yes**	No	Subject	2
10	The director that the actor visited wanted to discuss some minor edits.	Did the actor want to discuss the edits?	Yes	**No**	Object	1

10	The actor that visited the director demanded a role in the movie.	Did the director make demands?	Yes	**No**	Subject	1
10	The actor that the director visited demanded a role in the movie.	Did the director make demands?	Yes	**No**	Object	2
10	The director that visited the actor wanted to discuss some minor edits.	Did the actor want to discuss the edits?	Yes	**No**	Subject	2
11	The student that the professor criticized read the chapter after the lecture.	Did the student read after the lecture?	**Yes**	No	Object	1
11	The professor that criticized the student blushed and turned away very quickly.	Did the professor blush and turn away?	**Yes**	No	Subject	1
11	The professor that the student criticized blushed and turned away very quickly.	Did the professor blush and turn away?	**Yes**	No	Object	2
11	The student that criticized the professor read the chapter after the lecture.	Did the student read after the lecture?	**Yes**	No	Subject	2
12	The pilot that the flight attendant complimented asked for a simple favor.	Did the pilot compliment the flight attendant?	Yes	**No**	Object	1
12	The flight attendant that complimented the pilot feared flying before this job.	Did the pilot compliment the flight attendant?	Yes	**No**	Subject	1
12	The flight attendant that the pilot complimented feared flying before this job.	Did the flight attendant compliment the pilot?	Yes	**No**	Object	2
12	The pilot that complimented the flight attendant asked for a simple favor.	Did the flight attendant compliment the pilot?	Yes	**No**	Subject	2
13	The policeman that the burglar scared carried a flashlight and a weapon.	Did the burglar scare the policeman?	**Yes**	No	Object	1
13	The burglar that scared the policeman lived in a neighborhood across town.	Did the burglar scare the policeman?	**Yes**	No	Subject	1
13	The burglar that the policeman scared lived in a neighborhood across town.	Did the policeman scare the burglar?	**Yes**	No	Object	2
13	The policeman that scared the burglar carried a flashlight and a weapon.	Did the policeman scare the burglar?	**Yes**	No	Subject	2
14	The soldier that the civilian assisted received a medal from the army.	Did the civilian receive a medal from the army?	Yes	**No**	Object	1
14	The civilian that assisted the soldier died many years after the war.	Did the soldier die many years after the war?	Yes	**No**	Subject	1
14	The civilian that the soldier assisted died many years after the war.	Did the soldier die many years after the war?	Yes	**No**	Object	2
14	The soldier that assisted the civilian received a medal from the army.	Did the civilian receive a medal from the army?	Yes	**No**	Subject	2

15	The nurse that the doctor ignored risked the patient's life during surgery.	Did the doctor ignore the nurse?	**Yes**	No	Object	1
15	The doctor that ignored the nurse drove a red convertible to work.	Did the doctor ignore the nurse?	**Yes**	No	Subject	1
15	The doctor that the nurse ignored drove a red convertible to work.	Did the nurse ignore the doctor?	**Yes**	No	Object	2
15	The nurse that ignored the doctor risked the patient's life during surgery.	Did the nurse ignore the doctor?	**Yes**	No	Subject	2
16	The psychologist that the client confronted realized the mistake and later apologized.	Did the client apologize later?	Yes	**No**	Object	1
16	The client that confronted the psychologist wrote an angry letter that night.	Did the psychologist write an angry letter?	Yes	**No**	Subject	1
16	The client that the psychologist confronted wrote an angry letter that night.	Did the psychologist write an angry letter?	Yes	**No**	Object	2
16	The psychologist that confronted the client realized the mistake and later apologized.	Did the client apologize later?	Yes	**No**	Subject	2
17	The quarterback that the linebacker hated played a mean spirited practical joke.	Did the linebacker hate the quarterback?	**Yes**	No	Object	1
17	The linebacker that hated the quarterback signed a new three year contract.	Did the linebacker hate the quarterback?	**Yes**	No	Subject	1
17	The linebacker that the quarterback hated signed a new three year contract.	Did the quarterback hate the linebacker?	**Yes**	No	Object	2
17	The quarterback that hated the linebacker played a mean spirited practical joke.	Did the quarterback hate the linebacker?	**Yes**	No	Subject	2
18	The director that admired the dancer spent every evening at the theater.	Did the dancer admire the director?	Yes	**No**	Object	1
18	The dancer that admired the director worked at a school in Chicago.	Did the director admire the dancer?	Yes	**No**	Subject	1
18	The dancer that the director admired worked at a school in Chicago.	Did the dancer admire the director?	Yes	**No**	Object	2
18	The director that the dancer admired spent every evening at the theater.	Did the director admire the dancer?	Yes	**No**	Subject	2
19	The sheriff that the cowboy met smelled like an old medicine bottle.	Did the cowboy meet the sheriff?	**Yes**	No	Object	1
19	The cowboy that met the sheriff wore a leather vest and hat.	Did the cowboy meet the sheriff?	**Yes**	No	Subject	1
19	The cowboy that the sheriff met wore a leather vest and hat.	Did the sheriff meet the cowboy?	**Yes**	No	Object	2
19	The sheriff that met the cowboy smelled like an old medicine bottle.	Did the sheriff meet the cowboy?	**Yes**	No	Subject	2

20	The swimmer that the lifeguard encountered disliked swimming in the public pool.	Did the swimmer like swimming in the public pool?	Yes	**No**	Object	1
20	The lifeguard that encountered the swimmer had been out in the sun.	Was the lifeguard in the shade?	Yes	**No**	Subject	1
20	The lifeguard that the swimmer encountered had been out in the sun.	Was the lifeguard in the shade?	Yes	**No**	Object	2
20	The swimmer that encountered the lifeguard disliked swimming in the public pool.	Did the swimmer like swimming in the public pool?	Yes	**No**	Subject	2
21	In the book, the kitten was hugged by the bunny early morning.	Did the bunny hug the kitty?	Yes	No	Passive	1
21	In the book, the kitten was hugged by the bunny early morning.	Did the bunny hug the kitty?	Yes	No	Passive	2
22	Last month, the turtle was saved by the dolphin during the hurricane.	Did the hurricane happen last year?	Yes	No	Passive	1
22	Last month, the turtle was saved by the dolphin during the hurricane.	Did the hurricane happen last year?	Yes	No	Passive	2
23	On Thursday, the mailman was stopped by the policeman near the park.	Did the policeman stop the mailman?	Yes	No	Passive	1
23	On Thursday, the mailman was stopped by the policeman near the park.	Did the policeman stop the mailman?	Yes	No	Passive	2
24	Yesterday morning, the nurse was helped by the cowboy in ripped jeans.	Did the cowboy help the nurse last week?	Yes	No	Passive	1
24	Yesterday morning, the nurse was helped by the cowboy in ripped jeans.	Did the cowboy help the nurse last week?	Yes	No	Passive	2
25	Every morning, the prince was sung to by the queen before breakfast.	Did the queen sing to the prince?	Yes	No	Passive	1
25	Every morning, the prince was sung to by the queen before breakfast.	Did the queen sing to the prince?	Yes	No	Passive	2
26	Late Saturday, the clown was checked by the doctor after the show.	Did the doctor check the clown on Friday?	Yes	No	Passive	1
26	Late Saturday, the clown was checked by the doctor after the show.	Did the doctor check the clown on Friday?	Yes	No	Passive	2
27	After the accident, the lady was carried by a man to safety.	Did the man carry the lady after the accident?	Yes	No	Passive	1
27	After the accident, the lady was carried by a man to safety.	Did the man carry the lady after the accident?	Yes	No	Passive	2
28	Every night, the princess was read to by the king before bed.	Did the princess read to the king?	Yes	No	Passive	1
28	Every night, the princess was read to by the king before bed.	Did the princess read to the king?	Yes	No	Passive	2
29	On Friday, the boy was surprised by his grandmother with a present.	Did the grandmother surprise the boy with a present?	Yes	No	Passive	1

29	On Friday, the boy was surprised by his grandmother with a present.	Did the grandmother surprise the boy with a present?	Yes	No	Passive	2
30	Late afternoon, the mouse was splashed by the goose near the swing.	Did the goose splash the mouse in front of the house?	Yes	No	Passive	1
30	Late afternoon, the mouse was splashed by the goose near the swing.	Did the goose splash the mouse in front of the house?	Yes	No	Passive	2
31	Usually, the pilot was listened to by the astronaut during their conversations.	Did the astronaut listen to the pilot during their conversations?	Yes	No	Passive	1
31	Usually, the pilot was listened to by the astronaut during their conversations.	Did the astronaut listen to the pilot during their conversations?	Yes	No	Passive	2
32	On Sunday, the wolf was kicked by the zebra during the performance.	Did the wolf kick the zebra?	Yes	No	Passive	1
32	On Sunday, the wolf was kicked by the zebra during the performance.	Did the wolf kick the zebra?	Yes	No	Passive	2
33	In the cartoon, the sheep was tickled by the piglet wearing shoes.	Did the piglet wear shoes?	Yes	No	Passive	1
33	In the cartoon, the sheep was tickled by the piglet wearing shoes.	Did the piglet wear shoes?	Yes	No	Passive	2
34	Unexpectedly, the monkey was bumped by the squirrel during the show's rehearsal.	Did the monkey bumped the squirrel?	Yes	No	Passive	1
34	Unexpectedly, the monkey was bumped by the squirrel during the show's rehearsal.	Did the monkey bumped the squirrel?	Yes	No	Passive	2
35	Later that evening, the wizard was met by the cook before dinner.	Did the wizard and the cook meet before dinner?	Yes	No	Passive	1
35	Later that evening, the wizard was met by the cook before dinner.	Did the wizard and the cook meet before dinner?	Yes	No	Passive	2
36	During a game of hide-and-seek, the sister was found by her brother.	Did the brother hide?	Yes	No	Passive	1
36	During a game of hide-and-seek, the sister was found by her brother.	Did the brother hide?	Yes	No	Passive	2
37	After dusk, the tiger was chased by the lion into the jungle.	Did the tiger run into the jungle?	Yes	No	Passive	1
37	After dusk, the tiger was chased by the lion into the jungle.	Did the tiger run into the jungle?	Yes	No	Passive	2
38	In the zoo, the bear was smeared by the elephant with mud.	Did the bear smear the elephant?	Yes	No	Passive	1
38	In the zoo, the bear was smeared by the elephant with mud.	Did the bear smear the elephant?	Yes	No	Passive	2
39	Last week, the donkey was greeted by the lamb near the pond.	Did the lamb greet the donkey near the pond?	Yes	No	Passive	1
39	Last week, the donkey was greeted by the lamb near the pond.	Did the lamb greet the donkey near the pond?	Yes	No	Passive	2

40	Early evening, the cow was pushed by the horse in the meadow.	Did the cow push the horse?	Yes	No	Passive	1
40	Early evening, the cow was pushed by the horse in the meadow.	Did the cow push the horse?	Yes	No	Passive	2
41	I went to the store and bought milk, eggs, and green beans.	Did I go to the library?	Yes	No	Active	1
41	I went to the store and bought milk, eggs, and green beans.	Did I go to the library?	Yes	No	Active	2
42	I like to drink coffee and eat oatmeal every morning for breakfast.	Do I like to eat oatmeal for breakfast?	Yes	No	Active	1
42	I like to drink coffee and eat oatmeal every morning for breakfast.	Do I like to eat oatmeal for breakfast?	Yes	No	Active	2
43	He went for a run to get a breath of fresh air.	Did he go swimming?	Yes	No	Active	1
43	He went for a run to get a breath of fresh air.	Did he go swimming?	Yes	No	Active	2
44	The angry dog and cat were fighting for the same old toy.	Did the animals fight for the same old toy?	Yes	No	Active	1
44	The angry dog and cat were fighting for the same old toy.	Did the animals fight for the same old toy?	Yes	No	Active	2
45	She plays soccer every single day after school with the same friends.	Does she play basketball after school with friends?	Yes	No	Active	1
45	She plays soccer every single day after school with the same friends.	Does she play basketball after school with friends?	Yes	No	Active	2
46	He had some homework to do after playing outside in the rain.	Did it rain outside?	Yes	No	Active	1
46	He had some homework to do after playing outside in the rain.	Did it rain outside?	Yes	No	Active	2
47	She was tired after working late at night for three straight days.	Did she finish her work early?	Yes	No	Active	1
47	She was tired after working late at night for three straight days.	Did she finish her work early?	Yes	No	Active	2
48	Jack loves taking morning walks to start off a brand new day.	Does Jack love morning walks?	Yes	No	Active	1
48	Jack loves taking morning walks to start off a brand new day.	Does Jack love morning walks?	Yes	No	Active	2
49	Cookies and cream, strawberry, and chocolate are my favorite ice cream flavors.	Is the mint ice cream flavor my favorite?	Yes	No	Active	1
49	Cookies and cream, strawberry, and chocolate are my favorite ice cream flavors.	Is the mint ice cream flavor my favorite?	Yes	No	Active	2
50	I went to the big city to explore the beautiful tall buildings.	Did I go to the big city?	Yes	No	Active	1
50	I went to the big city to explore the beautiful tall buildings.	Did I go to the big city?	Yes	No	Active	2
51	She walks thirty minutes to work every day to get more exercise.	Does she drive to work?	Yes	No	Active	1
51	She walks thirty minutes to work every day to get more exercise.	Does she drive to work?	Yes	No	Active	2
52	Sarah's hobbies are reading, watching movies, and playing sports at the park.	Is reading one of Sarah's hobbies?	Yes	No	Active	1

52	Sarah's hobbies are reading, watching movies, and playing sports at the park.	Is reading one of Sarah's hobbies?	Yes	No	Active	2
53	I quickly ran to math class because I was ten minutes late.	Was I late to my English class?	Yes	No	Active	1
53	I quickly ran to math class because I was ten minutes late.	Was I late to my English class?	Yes	No	Active	2
54	I love going to the summer beach to make big sand castles.	Do I build sand castles on the beach?	Yes	No	Active	1
54	I love going to the summer beach to make big sand castles.	Do I build sand castles on the beach?	Yes	No	Active	2
55	I always have to take long naps after coming home from school.	Do I have play outside after coming home from school?	Yes	No	Active	1
55	I always have to take long naps after coming home from school.	Do I have play outside after coming home from school?	Yes	No	Active	2
56	The cute rabbit was eating all of the carrots in the garden.	Was the rabbit eating carrots?	Yes	No	Active	1
56	The cute rabbit was eating all of the carrots in the garden.	Was the rabbit eating carrots?	Yes	No	Active	2
57	I made a huge snowman with the snow in the front yard.	Did I make a small snowman?	Yes	No	Active	1
57	I made a huge snowman with the snow in the front yard.	Did I make a small snowman?	Yes	No	Active	2
58	I like tomato, lettuce, and yellow peppers in my salad for lunch.	Do I like tomatoes, lettuce, and yellow peppers in my salad?	Yes	No	Active	1
58	I like tomato, lettuce, and yellow peppers in my salad for lunch.	Do I like tomatoes, lettuce, and yellow peppers in my salad?	Yes	No	Active	2
59	She went to sleep early because she needed to wake up early.	Did she go to sleep late?	Yes	No	Active	1
59	She went to sleep early because she needed to wake up early.	Did she go to sleep late?	Yes	No	Active	2
60	The freshly baked chocolate chip cookies were still very warm and chewy.	Were the cookies freshly baked?	Yes	No	Active	1
60	The freshly baked chocolate chip cookies were still very warm and chewy.	Were the cookies freshly baked?	Yes	No	Active	2
